# Keratosis obturans complicated with facial nerve palsy: a diagnostic dilemma^☆^

**DOI:** 10.1016/j.bjorl.2016.04.012

**Published:** 2016-05-24

**Authors:** Jeyasakthy Saniasiaya, Nik Adilah Nik Othman, Nur Hidayati Mohamad Pakarul Razy

**Affiliations:** a Universiti Sains Malaysia Health Campus, School of Medical Sciences, Department of Otorhinolaryngology, Head & Neck Surgery, Kelantan, Malaysia; b Universiti Sains Malaysia Health Campus, School of Health Sciences, Audiology Program, Kelantan, Malaysia; c Universiti Sains Malaysia Health Campus, School of Health Sciences, Department of Pathology, Kelantan, Malaysia

## Introduction

Keratosis obturans is estimated to occur among 4 to 5 patients among 1000 new otological cases.^1^ Typical clinical manifestations includes severe otalgia and hearing loss attributed by the accumulation of desquamated epidermal plug in ear canal. The epidermal plug formation may occur due to faulty migration or excessive production of epithelial cells, as proposed by Paparella and Shumrick.^2^ Ballooning or widening of bony external auditory canal can also specify keratosis obturans.^3^ For many years, keratosis obturans and external canal cholesteatoma were used interchangeably up until 1980 when Piepergedes and Behnke distinguished them as a separate entity.^4^ To our knowledge this is the first reported case of an unusual presentation of keratosis obturans causing unilateral facial palsy with no evidence of bony erosion.

## Case report

A previously healthy 22 year-old Malay gentleman presented to our clinic with a two-month history of worsening left otalgia with bloody otorrhea. There was also left sided reduced hearing followed by asymmetry on the left side of face for the past 1 week. Patient claimed that there was no recent or any recurrent upper respiratory tract infection prior to this.

Further history from patient revealed that he had similar complaints a year ago involving the right ear with no facial asymmetry. Patient was diagnosed with right aural polyp and polypectomy was done under local anaesthesia in another government hospital. However, patient defaulted his follow-up as the problem resolved completely.

**Figure 1:**
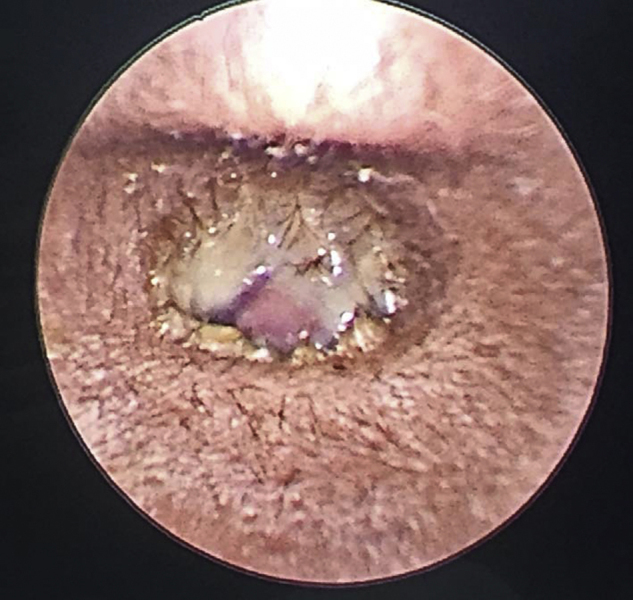
Polypoidal mass occluding left ear canal.

Upon examination, patient was comfortable, not septic looking and afebrile. Facial nerve examination revealed House–Brackmann Grade III lower motor neuron palsy as there was loss of left nasolabial fold and drooping of left angle of lip. Otoscopic examination revealed a polypoidal mass occluding the entire left ear canal covered with bloody, foul smelling otorrhea (Fig. 1). Right ear canal examination was normal with an intact tympanic membrane. Oropharynx examination was unremarkable and neck nodes were not palpable. Nasoendoscopy revealed mild adenoid enlargement with no signs of active infection. All other cranial nerves were intact and no other neurological deficit was evident. Systemic examination was normal. Tuning fork test revealed left conductive hearing loss. Full blood count and electrolytes were within normal range. Preliminary diagnosis of left aural polyp with facial nerve palsy grade III was made with a differential diagnosis of external canal cholesteatoma. Patient was started with intravenous ciprofloxacin with tapering dose of prednisolone. Subsequent day, facial nerve palsy was noted to improve demonstrating facial nerve palsy House–Brackmann grade II.

**Figure 2:**
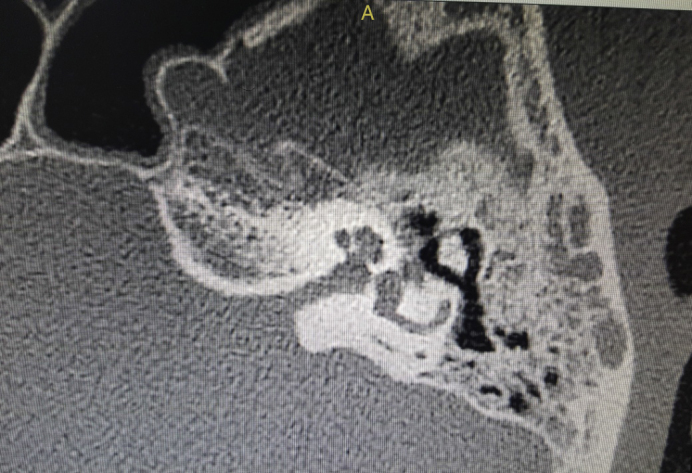
Inflammation over tympanic segment of facial nerve.

A high-resolution computer tomography (HRCT) scan of temporal was obtained, which revealed non-enhancing soft-tissue mass occupying left external auditory canal, left Prussak's space and middle ear with erosion of left scutum. The left inner ear was intact with normal right ear structures. There was no evidence of facial canal dehiscence or erosion; however signs of inflammation over the tympanic segment of facial canal were noted (Fig. 2).

**Figure 3:**
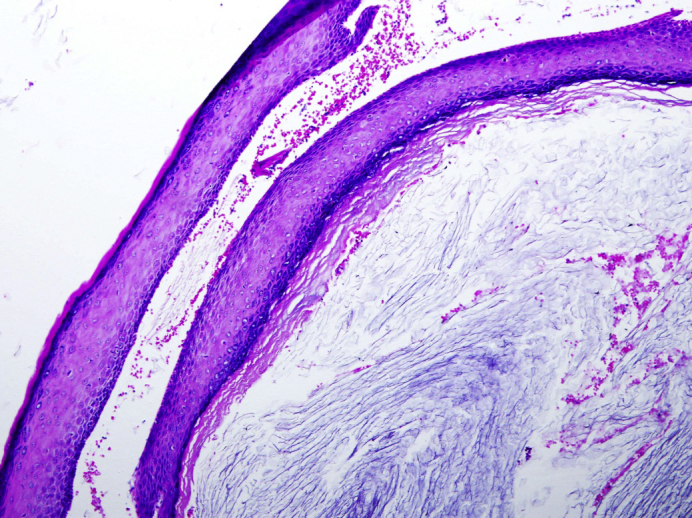
Cyst wall containing lamellated keratin flakes.

Patient underwent microscope-guided examination under anaesthesia which revealed polypoidal mass occupying entire lateral third of ear canal with whitish keratin-like debris noted medial to the mass. Polypectomy and aural toileting was commenced. As tympanic membrane appeared bulging, myringotomy and grommet insertion was performed. Histopathologic examination revealed cyst wall covered with stratified squamous epithelium containing lamellated keratin flakes suggestive of keratosis obturans (Fig. 3) Post-operatively patient was well. He was discharged home following day as the facial asymmetry improved with only slight weakness noted upon close inspection with no other accompanying complications. Oral ciprofloxacin, tapering dose of prednisolone and ofloxacin ear drops was prescribed for 1 week duration upon discharge. He was given a one week appointment which he defaulted.

## Discussion

Keratosis obturans can be of two types: Inflammatory type or silent type.^1^ It has been proposed that the inflammatory type occurs secondary to an acute infection, for instance viral infection whereby the inflammatory process may temporarily alter the epithelial migration. The inflammatory type may be cured after removal of the keratin. As for the silent type, it is due to an abnormal separation of the keratin causing the disease to progress continuously even after the first removal thus, requiring continuous aural toileting. Our patient may be categorised into the inflammatory type of keratosis obturans.

Keratosis obturans usually affects younger age group, occurs bilaterally and manifests as severe otalgia, conductive hearing loss and widened ear canal.^5^ Otorrhea is considered a rare presentation.^5^ Seventy seven percent of children and twenty present of adults have an associated sinusitis and bronchiectasis which is due to reflex sympathetic autonomic activation leading to excessive cerumen secretion thus epidermal plug formation.^6^

Computer tomography typically demonstrates soft tissue plug in bilateral external ear canal with evidence of ballooning of the osseous part. In our patient, his main complaint was severe otalgia and otorrhea which was followed by unilateral facial weakness and hearing loss. Although rare, few cases of keratosis obturans, presenting with facial weakness have been reported.^7^, ^8^ Facial nerve palsy following keratosis obturans are caused by bony erosion,^7^, ^8^ which may be due to the pressure effect exerted by keratin mass in the external canal.^9^ In our patient, albeit no evidence of bony erosion in the HRCT temporal, pressure exerted by acute inflammation may have caused the facial nerve palsy which was supported by complete resolution after commencement of antibiotics and surgical removal of the ear mass.

Clinical differential diagnosis for mass in aural canal with facial nerve palsy includes external auditory canal cholesteatoma, neoplasms of external canal and malignant otitis externa. It is important to distinguish the diagnosis, as management of each of the differential diagnosis is notably different. Hence, thorough and detailed history, physical examination, radiological examination and most importantly histopathological examination is crucial prior to a diagnosis. Histopathological examination of the biopsied or excised mass is the main modality of diagnosis, more so when there is an atypical or rare presentation.

As for management, keratosis obturans usually requires frequent aural toileting to remove the keratin plug and topical medication. This may be done under general anaesthesia especially among non-cooperative patients. Split skin graft and canalplasty method have also been reported for refractory keratosis obturans.^10^ As for cases of keratosis obturans complicated with facial nerve palsy, steroids may be prescribed along with antibiotics if there is precipitating infection.

## Conclusion

Albeit commonly regarded benign, keratosis obturans may result in devastating complications including facial nerve palsy as in our patient. Hence, high clinical suspicion and prompt management among clinicians are of dire importance as absence of typical clinical features usually lands both the attending physician and patient in dilemma.

## Conflicts of interest

The authors declare no conflicts of interest.
